# Ultrasound-guided injection technique of the equine cervical nerve roots

**DOI:** 10.3389/fvets.2022.992208

**Published:** 2022-10-26

**Authors:** Gregoire Fouquet, Ghazanfar Abbas, Jessica P. Johnson, Endrigo Pompermayer, Camille Harel, Eman Aldous, Sarah Puchalski, Florent David

**Affiliations:** ^1^Equine Veterinary Medical Center, Member of Qatar Foundation, Doha, Qatar; ^2^Puchalski Equine Diagnostic Imaging Inc., Petaluma, CA, United States

**Keywords:** radiculopathy, cervical nerve root, ultrasound-guided, horse, equine, computed tomography, neck, injection

## Abstract

Radiculopathy in horses is often a diagnosis of exclusion because of the non-specific clinical signs related to neck pain and possible forelimb lameness. There are no reported treatment options in the equine veterinary literature. The purpose of the study was to describe an ultrasound-guided injection of the cervical nerve root C3 to C8, to evaluate accuracy, time and safety and to anticipate possible complications on clinical cases. Under general anesthesia and with ultrasound guidance, five horses were injected from C3 to C8 with 1.5mL mix of contrast and latex. Immediately after euthanasia, the necks were taken for CT examination and then dissection was performed 3 days later. Data regarding the accuracy of injection, the presence of injectate in the nerve root, vertebral vessel or vertebral canal were recorded from both CT and dissection. The time of injection and ability to visualize the nerve root prior to injection were also recorded. Out of 60 intended injections, 55 (CT images) and 57 (dissection) led to injectate deposited within the target zone with direct contact between contrast/latex and cervical nerve roots noted in 76.4% and 73.7%, respectively. Presence of contrast/latex injectate within nerves (≤11%), vertebral vessels (<4%) and canal (<4%) were rarely encountered. No variation on success rate or safety noted based on the site of injection. The technique described has excellent accuracy, with injectate deposition in direct contact (≈75%) or close vicinity (≈25%) of C3-C8 cervical nerve roots. Injectate diffusion is likely to further improve success rate. Rare presence of injectate within nerve/sheath, vertebral vessels/canal along with diffusion warrants caution when performing this procedure in clinical cases.

## Introduction

Ultrasound-guided (UG) injections of the cervical articular process joints (APJ) is a procedure commonly performed in horses to alleviate neck pain associated with APJ osteoarthritis (1). However, there is evidence showing that horses could also suffer from cervical nerve root radiculopathy, which has been well-described in humans and dogs (2, 3). Radiculopathy has been diagnosed in horses with the help of advanced imaging or at post mortem with histopathology of the cervical nerve roots (4–6). Possible clinical signs observed in horses are a combination of neck pain and stiffness, muscle atrophy, hypo- or hypersensitivity, sweat patches and hopping type forelimb lameness (4). These signs may suggest neck pathology, though are non-specific which makes a definitive diagnosis of radiculopathy difficult.

Etiopathology of cervical radiculopathy in horses has only been associated with compression of the nerve root secondary to narrowing of the intervertebral foramen (IVF) and remodeling of the APJ due to osteoarthritis (4–6). Diagnosis in live horses can only be done with advanced imaging but it is not routinely performed because of the current difficulty to image the caudal part of the neck.

After exiting the IVF, the cervical nerve roots divide into a dorsal (primarily sensory and autonomic fibers) and ventral (primarily motor and autonomic fibers) branch. The ventral branches of the cervical nerve roots 6 to 8 (C6–C8) form the brachial plexus with the first thoracic nerves, T1 and T2, which then innervates the forelimb. This explains why compression of C6–C8 can lead to forelimb lameness. Lameness due to radiculopathy can present with various degrees of severity and can be intermittent depending on factors such as being on one rein, head position, or work under saddle (4). Another clinical feature described is the nerve root signature posture, with horses keeping the affected forelimb in a semi-flexed position and being reluctant to bear weight. This sign is relatively rare but has been reported in horses and is recognized as a clinical sign of nerve impingement or entrapment in dogs (4, 7, 8).

In other species, radiculopathy is commonly treated with perineural corticosteroid injection of the cervical nerve roots, either within the nerve root or around the nerves when they exit the IVF (3, 9). These injections are performed with the aid of imaging modalities such as fluoroscopy or ultrasound, with the latter being more commonly performed to further reduce the risk of inadvertent penetration of important structures (vertebral or radicular arteries) present in the IVF leading to severe complications such as brain and/or spinal cord infarction, paralysis, seizures, and death (9, 10). Multiple cadaveric studies have been performed in horses aiming at developing UG injection techniques of the cervical nerve roots and showed promising results (11–14). The study performed by Wood et al. had a 73% success rate with direct contact between the injectate and the ventral ramus of the targeted cervical nerve root (13). This study focused on injecting C5 and C6 and the skin of the cadavers was removed prior to performing the injections, which altered the conditions compared to live horses (13).

The study objectives were to describe an UG injection technique of the equine cervical nerve roots 3 to 8, to evaluate accuracy, time necessary to perform UG injections and safety in anesthetized and asymptomatic horses, and to anticipate on possible complications in clinical cases.

## Materials and methods

### Pilot study

To better understand the IVF anatomy, five horses presented to the Equine Veterinary Medical Center—a member of Qatar Foundation and who were euthanized for reasons unrelated to neck problems were dissected between 24 and 72 h after death. Both sides of the neck were dissected and the cervical nerve roots C2 to C8 exposed. Their location within the IVF and spatial relationship with the APJ, the vertebral artery, vein and collateral vessels were noted.

As a next step, two sedated adult horses were evaluated with ultrasound imaging (Logic E9 XDclear 2.0; GE Healthcare, Chicago, IL, United States) on repeated occasions until the ultrasonographic anatomy of the IVF and associated structures (cervical nerve roots C2 to C8 on each side of the neck and vertebral vessels) was fully understood. The ability to see the ventral ramus of each nerve and to locate the vertebral vessels with confidence and at all sites was evaluated using 2D US mode and color Doppler using 2 transducers (C1–6 MHz convex and ML6–15 MHz linear; GE Healthcare, Chicago, IL, United States).

### UG injection technique

Five Arabian gray mares (age range 20–22 years old) donated to the Equine Veterinary Medical Center—a member of Qatar Foundation for chronic health issues unrelated to neck problems were used for the study. A catheter was placed in the left or right jugular vein, and the horses were sedated with Xylazine (1.1 mg/kg IV; Thiazine 100 injection, Ceva Animal Health Pty Ltd, Australia) before induction of general anesthesia with Ketamine (2.5 mg/kg IV; Ketamine 10%, Alfasan, Netherlands). The horses were then intubated before being moved to a surgery table and placed in lateral recumbency, either right or left determined randomly. During the whole procedure, the horses were maintained under general anesthesia with Isoflurane (Fluriso, MWI Animal Health, Idaho, United States) and vital parameters were monitored. The upper front leg was retracted caudally as far as possible to maximize exposure to C7–T1 IVF and secured in place with a rope. The neck was palpated to identify the second vertebra (axis) and clipping/preparation of the skin from the axis to the cranial aspect of the scapula took place. The ultrasound machine (Logic E9 XDclear 2.0; GE Healthcare, Chicago, IL, United States) was placed on the dorsal neck side with the screen facing the operator, who was standing on the ventral neck side. After palpation of the axis' sagittal ridge, coupling gel was applied and a 6 MHz curvilinear macroconvex transducer (C1-6 MHz convex; GE Healthcare, Chicago, IL, United States) was used to identify C2–C3 APJ with the probe oriented along a dorsal-ventral axis (vertical position in a standing horse).

**Figure 1:**
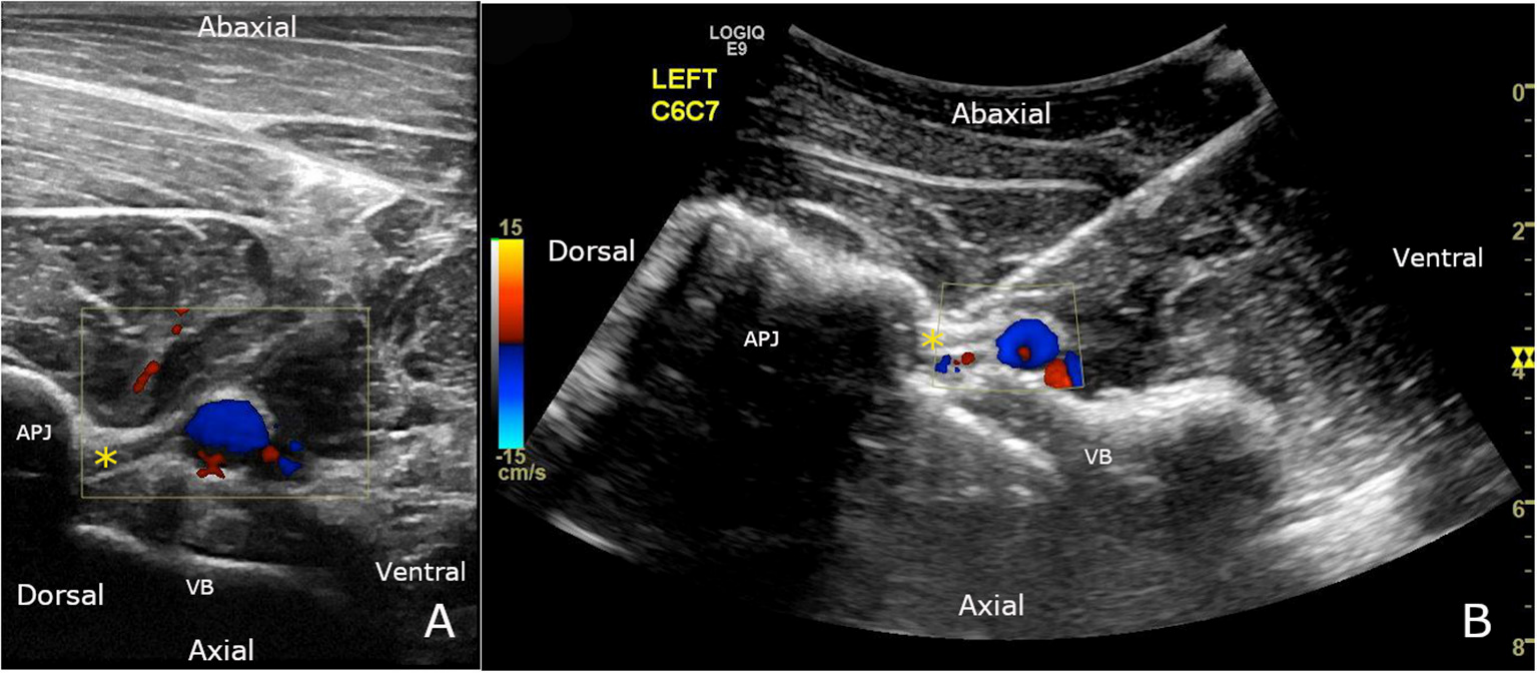
**(A)** Ventral branch of C7 (*) can be seen crossing obliquely over and immediately dorsal to the vertebral vessels, using a 12 MHz linear transducer. The location of the vertebral vessels and collaterals is confirmed using color Doppler mode. **(B)** C7 nerve root ultrasound-guided injection, using a 6 MHz curvilinear transducer. The spinal needle is inserted until the tip is visualized in contact with the nerve (*). The final approach is performed with color Doppler to ensure there is no blood flow where the needle tip is inserted. APJ, articular process joint; VB, vertebral body.

The transducer was then displaced ventrally to focus on the C2–C3 IVF zone demarcated by the cranial articular process of C3 dorsally and the caudal aspect of the vertebral body of C2 ventrally, with the vertebral vessels (artery and vein) running abaxial to the osseous structures and imaged in transverse section. The gain and focal zone were adjusted to obtain the best image quality. The transducer was displaced from cranial to caudal to look for the ventral ramus of C3 and to clearly identify the vertebral vessels and their collaterals using 2D and color Doppler mode. The vertebral vessels were identified first then the cervical nerve roots found running just dorsal to it. On 2D ultrasound, the ventral ramus of the cervical nerve was represented by a 2–5 mm linear hypoechogenic to echogenic structure delineated by two parallel hyperechogenic borders, as previously described by Alexander and Dobson (15). To best visualize the ventral ramus of the cervical nerve, a final adjustment of the transducer orientation from the initial dorsal-ventral axis was often needed by rotating the transducer by 5–25 degrees clockwise on the right side of the neck or by 5–25 degrees anti-clockwise on the left side, with the more rotation needed on the caudal neck. Images (Figure 1A) and videos were recorded during this process. Once identified and/or satisfied with the positioning, the operator inserted an 18 G, 9 cm spinal needle immediately ventral to the transducer. The needle was advanced through the neck muscles and the angle of approach adjusted to reach the target. For the final approach, the needle was guided slowly until the tip was placed dorsal to the vertebral artery, right against the ventral ramus of C3. Images and videos were also recorded during this process.

Once satisfied with the positioning and to ensure no further movement, the needle was held in place close to the skin by the operator's assistant who removed the stylet and injected a mixture of 0.5 ml of colored latex and 1 ml of radiographic contrast (300 mg/ml organically bound iodine, Isovue-300, Bracco Diagnostics, Germany).

The same procedure (Figure 1B) was repeated from C4 to C8 (ultrasound field of view depth adjusted for each site; new needle used at each site) on the same side and then on C3 to C8 on the other side after repositioning the horse. All injections were performed by the same operator who had experience with UG injections.

For each injection, the time was recorded from needle insertion through the skin to removal of the stylet and any observations or difficulties were recorded.

The horse was euthanized at the end of the procedure with a lethal dose of embutramide, mebezonium and tetracaine combination (T61; MSD Animal Health, Kenilworth, NJ, United States) administered intravenously.

### Accuracy of injection analysis via CT scan

Immediately after euthanasia, the head and neck were separated from the trunk at the level of T2-T3 and ribs 1 and 2 were transected. The specimens were placed on the in-built CT table and a CT scan (Somatom Definition AS, 128-slice; Siemens, Munich, Germany) of the neck was performed. One-millimeter helical images processed by using a high-frequency convolution kernel were acquired (parameters 35 Ma, 140 KV, 0.6 mm slice thickness) in left lateral recumbency. The scan quality was ascertained before the neck was removed from the gantry and the final images interpreted by a board-certified radiologist. Data recorded included the presence of neck pathology, contrast material identified at the nerve root and the distance between contrast and the nerve root if there was no direct contact, the presence of contrast within the vertebral vessels and the vertebral canal. The nerve roots could not be observed on the CT images so the presence of contrast material identified at the nerve roots or the distance measured in cases of no direct contact were based on the expected anatomical location of the nerve roots within the IVF.

### Accuracy of injection analysis via dissection

The necks were then placed in a cooler for 72 h before dissection to allow the latex to harden. The skin and superficial muscles were removed until the cervical transverse process, APJ, and the ventral branch of the cervical nerve roots were observed. The *intertransversarius* muscle was carefully dissected to expose the IVF and the latex balls. Data recorded were the presence of latex at the nerve root and the distance between the latex and the dorsal and ventral ramus if there was no direct contact to the nerve root, and the presence or absence of latex within the vertebral vessels. The cranial articular process of each cervical vertebra was then transected with an osteotome and removed to visualize the vertebral canal. Data recorded were the presence or absence of latex within the vertebral canal.

### Statistical analysis

For quantitative variables such as distances or time of injection, the median and interquartile range were provided as they were not normally distributed. To test the association between injection site and contact with the targeted cervical nerve root, a Cochran-Mantel-Haenszel test was used. This test took into account multiple measures for each subject. A Friedman's test for repeated measures was used to examine whether the distribution of the quantitative variables varied according to the injection site. This test was performed separately for the right and left sides, since Friedman's test can only incorporate one repeated factor at a time. The kappa coefficient was used to determine whether the injection sites that did not reach the target based on dissection results were the same as those that failed to reach the target based on CT results. A mixed logistic regression model was used to determine whether the chances of contact varied with the time of injection. The model included ID (the five subjects with repeated measures) as a random factor and time of injection as a fixed factor. To test the effect of experience, we replaced injection site with a number from 1 to 6 (nerve C3 being 1, C4 being 2 until C8 being 6) to reflect the gradual increase in experience with cervical nerve root UG injections. We used a mixed logistic regression model to determine whether the chances of success varied with the sequence of injections. The model included ID (the five subjects with repeated measures) as a random factor and sequence of injections (1 to 6) as a fixed factor. Statistical significance was set at *p* <0.05.

## Results

### Anatomic and ultrasonographic anatomic findings (pilot study)

After exiting the IVF, the cervical nerve root quickly divided into the dorsal and ventral branch. The dorsal branch ran just caudal to the articular process along with collateral blood vessels. The ventral branch ran on top of the vertebral artery and vein, in a craniocaudal and dorsoventral oblique direction. The vertebral arteries and veins passed through the vertebral foramen from vertebrae C2 to C6 but then beneath the transverse process of vertebra C7.

In standing horses, the IVF could be observed from C2 to C7 but there was difficulty getting a good image of the foramen at C7–T1 because of the scapula obstructing the view. The vertebral artery could always be observed and its position was confirmed with color Doppler mode but the vein was not always clearly visible, even with the help of color Doppler. It was noted that the ventral branches of the cervical nerves were more easily identified in the caudal part of the neck, as they were larger.

### UG injection—procedure findings

Of the 60 intended injections, only 59 were performed in the study. In one horse, the left C3 cervical nerve root was misidentified and C4 was injected first instead of C3, making the last injection on this side impossible (T1 nerve instead of C8).

The same ultrasonographic observations noted in the pilot study were made in anesthetized horses, with the ventral branch of the cervical nerve being observed in 89% of the sites in total and in 96.7% for C6 to C8 spinal cervical nerves.

Some blood was obtained twice during injections when the spinal needle stylet was removed, on one horse (#92,415) for C4 and C5 on the left side, resulting in the operator redirecting the needle before injection. In total, the needle was redirected in 6.8% (4/59) of the cases, two times because of inadvertent blood vessel penetration as described above, one time because the horse became too light under general anesthesia and moved slightly just before injection and one time because the operator hit the transverse process of the cranial cervical vertebra during the initial approach.

All injections were well–tolerated and the veterinarian performing anesthesia did not detect any vital parameter changes during the procedure. The mean injection time was 40s ± 23.25 and the time for injection did not vary significantly between the different sites (right side *p* = 0.94 and left side *p* = 0.83) and did not affect the success rate (*p* = 0.40).

### CT and dissection findings (Table 1)

**Figure 2:**
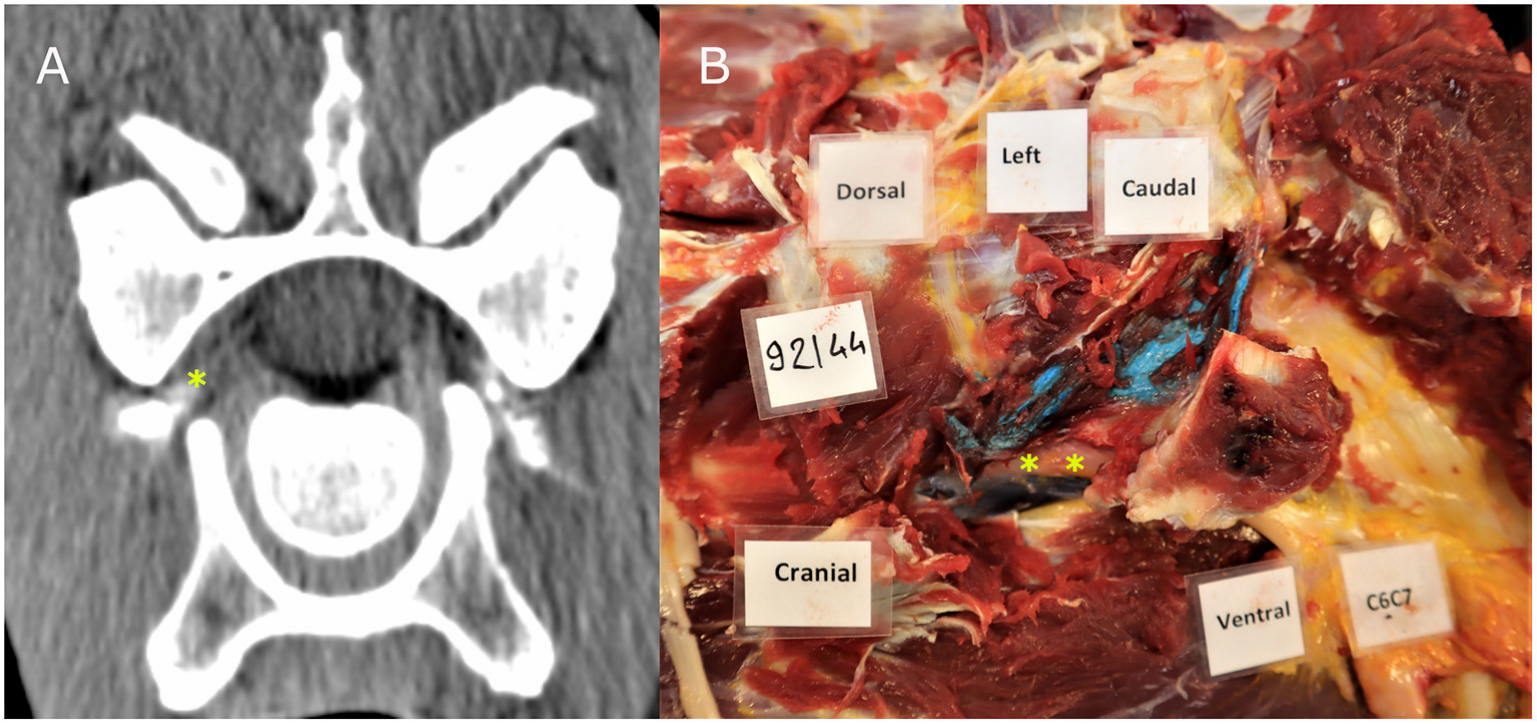
CT image **(A)** showing contrast material in the intervertebral foramen where the cervical nerve root C7 is located; Dissection image **(B)** showing direct contact between blue latex with the cervical nerve root at the level of C6–C7. The nerve root and ventral branch of C7 are indicated by a yellow asterisk.

On transverse, reformatted CT images, with the intervertebral foramen positioned in a symmetric manner, contrast media accumulation at the level of the lateral aspect of the intervertebral foramen outlining tubular filling defects was considered positive for direct contact between the injectate and the nerve roots. This was identified in 76.4% of the injections, based on CT findings (Figure 2A). Direct contact between latex and nerve root was noted in 73.7% of the injections, based on dissection findings (Figure 2B). There were 15 injections that did not result in injectate being present at the expected location of the cervical nerve root on CT findings and the same number did not have direct contact with the nerve root on dissection. The median distance (interquartile range Q3–Q1) between latex or contrast and the nerve root if direct contact was not achieved was 13 mm (IQR 4) on CT and 4 mm (IQR 3.5) on dissection.

**Table 1:** Cervical nerve root injection findings based on CT and dissection.

	**Number of injections retrieved**	**Direct contact between injectate and nerve root frequency (%)**	**Median (IQR) distance between injectate and nerve root if no direct contact (mm)**	**Injectate presence within vertebral vessels frequency (%)**	**Injectate noted in nerve sheath frequency (%)**	**Injectate noted in vertebral canal frequency (%)**
CT	55	76.4^*^	13 (4.0)	3.6 (vessel wall)	N/A	3.6
Dissection	57	Ventral and dorsal branches: 73.7 Dorsal branch: 98.3	4 (3.5)	0	11	1.7

* Direct contact was determined when contrast media accumulated around tubular filling defects in the anatomic location of the nerve roots, just lateral to the respective intervertebral foramen.

**Figure 3:**
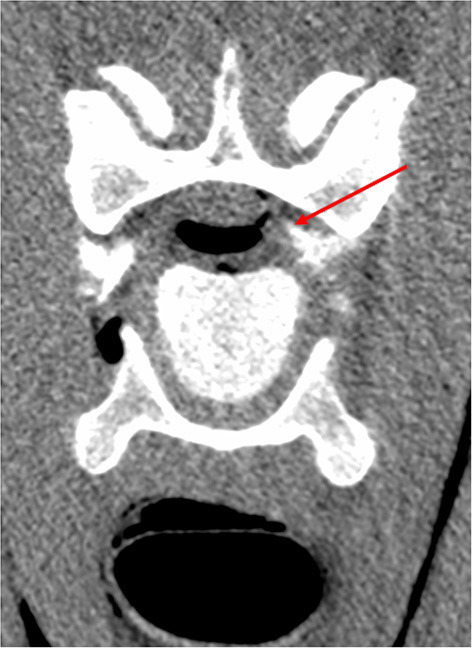
CT image showing contrast within the vertebral canal (red arrow) at the level of C6–C7.

**Figure 4:**
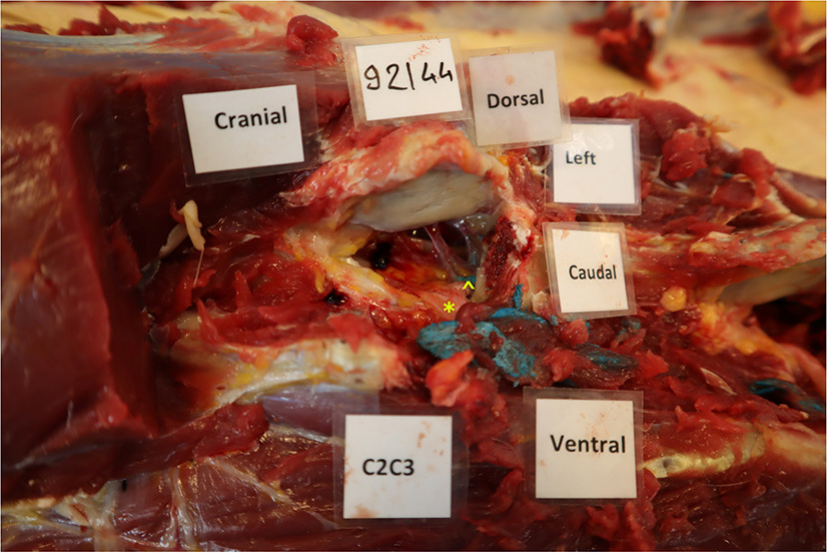
Presence of blue latex within the vertebral canal at the level of C3. The left cranial and abaxial articular process of the 3^rd^ cervical vertebra was transected with an osteotome to visualize the vertebral canal. In this case, the latex was in contact with the nerve root (*) but a small amount was also present within the vertebral canal (∧), but outside of the dura mater.

**Figure 5:**
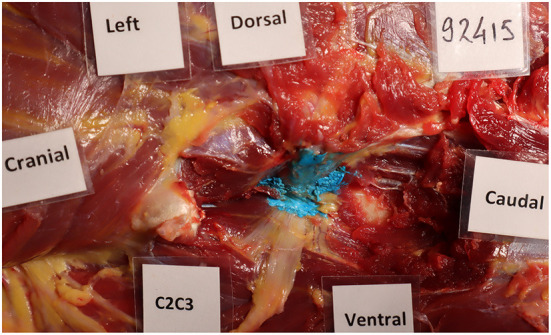
Presence of latex (blue) within the nerve sheath of C3.

A small amount of injectate was found in the vertebral canal in 3.6% (2/55) of the injections on CT (Figure 3) and 1.7% (1/57) on dissection (Figure 4). Latex was noted in the nerve sheath in 11% (6/57) of the injections, according to the dissection results (Figure 5). Contrast material was noted in the vertebral vessel wall in 3.6% (2/55) of the injections, based on CT results. However, no latex was noted during dissection within the vertebral vessels for any of the injections.

Based on statistical analysis, no correlation was found between the targeted cervical nerve root injected and the outcome. The success rate (*p* = 0.10 for dissection results and *p* = 0.62 for CT results), distance between the injectate and cervical nerve root (*p* = 0.55 for the right side and *p* = 0.22 for the left side based on dissection results; *p* = 0.23 for the right side and *p* = 0.51 for the left side based on dissection results) or risk of injection within the vessels, nerve sheath (*p* = 0.57) or vertebral canal was not affected by the site of injection. There was also no effect of a learning curve by the single operator doing the injections (*p* = 0.83).

Two injections out of 59 were not retrieved during dissection in one horse (#92,119) at the level of C4 on the left side and C3 on the right side of the neck. Four injections, including the two locations at which latex was not retrieved during dissection on the same horse mentioned above, were not identified on CT at the level of C3 and C4 on the left and right side. The horse on which the injections were not retrieved on dissection and on CT examination underwent a more extensive dissection. The vertebral veins and artery were dissected and the lumen opened along an extended distance. No latex material could be identified in the vertebral vessel lumen. As the vertebral artery is the closest from the ventral branch, the brain of this horse was also carefully examined to look for a cerebral latex embolism. Transverse serial sections (1 cm thick) of the brain were scrutinized and no latex material was found either. During dissection, a small hematoma was encountered around the point of injection on 2 occasions, corresponding to the same sites, C4 and C5 on the left side of horse #92,415, where blood was noted during the stylet removal and where the needle was repositioned prior to injection.

## Discussion

This study described and evaluated a novel UG injection technique of the cervical nerve roots C3 to C8 in 5 anesthetized horses, with the needle inserted ventral to a macroconvex transducer and guided toward the IVF. The needle tip was secured against the ventral branch of the cervical nerve (readily visible in 89% of the cases) and immediately dorsal to the vertebral artery as shown on color Doppler. This technique was highly reliable with an accuracy of injection of ~75% for direct contact between a very low volume injectate and the nerve root (and the remaining within <5 mm of nerve root on dissection).

The APJ and vertebral arteries were visualized and used as a landmark to identify the ventral branch of the cervical nerve root exiting the IVF and to guide the needle insertion. In 89% of injections, the ventral nerve root branch was localized before inserting the needle and it was found to be easier in the caudal part of the neck where the ventral branches of the cervical nerves are wider. This is in agreement with the study performed by Tourzot-Jourde et al. focusing on injecting C7 and C8 in cadavers, and where the ventral branches were seen in all cases (*n* = 10) (14). If not directly visualized, the main landmark used to guide the needle positioning in the present study was the vertebral artery at the caudal aspect of the IVF. Based on the pilot study findings, the ventral branch separates from the cervical nerve root and runs immediately dorsal to the vertebral blood vessels. Other studies have shown that direct visualization of the nerve is not necessary to obtain good results and other specific landmarks can be used such as immediately below the ventral margin of the cranial articular process (transducer parallel to the short axis of the neck) or the region between the APJ and the vertebral body (transducer parallel to the long axis of the neck) (12, 13). It is however highlighted that a good understanding of the anatomy and structures within and surrounding the IVF is required to perform this procedure successfully and safely (12, 13).

Based on the technique described in our study, UG injection of cervical nerve root is possible with very good results anticipated. Approximatively 75% of the injections resulted in direct contact between the low volume injectate (1.5 ml) and the nerve root while the remaining injections were within 5 mm of the nerve root visualized at dissection. These results are similar to what has been previously described by Wood et al. but lower than what was described in the studies by Cruz-Sanabria et al. and Tourzot-Jourde et al. (12–14). However, the latter two had small study samples (total number of injections 18 and 10, respectively) and the volume of injection in Tourzot-Jourde et al. study was much higher (volume of liquid injectate = 7 or 14 ml), which may have overestimated the accuracy of the injection (14). For the purpose of our study, it was decided to inject the cervical nerve root C3 to C8 even though clinical cases of cranial neck radiculopathy are likely to be less obvious than caudal neck radiculopathy (brachial plexus consists of C6 to T2 spinal nerve ventral rami) where performance-limiting gait abnormalities may be observed. The objectives were to increase the number of injections performed on each horse and also to assess if the results of the UG injection technique described herein were consistent along the neck, despite significant anatomical variations at the level of C8. The injection technique has been shown to be repeatable and the location of injection did not influence the success rate or safety of the procedure. Our study has the advantage of precisely assessing accuracy of injection because of the very low volume of liquid injectate used (1.5 ml total volume including 1 ml of latex). In addition, horses affected by radiculopathy are likely to be injected with larger volumes. This is likely to increase the spreading effect and should result in better success rates, as shown by Tourzot-Jourde and collaborators (14).

Safety is a major factor to take into consideration when developing and testing an injection technique in the close vicinity of the IVF. This is particularly relevant because risks associated with incorrect needle positioning or injection can lead to fatal complications due to the structures present within or surrounding the IVF (vertebral or radicular arteries, spinal cord and vertebral canal) (9, 16). Also, some anatomic variations may be encountered secondary to the development of APJ osteoarthritis, modifying the usual landmarks. Dissection revealed injectate within the nerve sheath in 11% of the injection sites. This result highlights the precision of our injection technique to place the needle tip in contact with the nerve. In a sedated and clinical case, a similar needle placement is not likely to lead to a fatal complication but will possibly cause a reaction from the horse, which could possibly lead to a safety issue for the horse itself, the personnel performing the injection or holding the horse and the equipment. Keeping a 3–5 mm distance between the needle tip and the ventral branch of the nerve would be advisable as a safety margin to avoid this problem in clinically sedated cases where visualization of the nerve would be good. For the clinical cases where the ventral branch cannot be identified properly, this problem may occur during the initial needle insertion, or if the horse moves or if the operator inadvertently pushes the needle whilst the stylet is removed. To minimize this risk, we would advise to keep the horse at a good level of sedation with the head supported on a fixed surface (head support), to have the skin desensitized by a local anesthetic injection, and to have an assistant helping the operator to remove the stylet while the needle is kept steady at the skin level and during the injection. One of the major safety concerns is the penetration or injection of material intra-arterially, which is likely the main cause of severe complications including death secondary to cervical nerve root injections in humans (16–18). During our study, no complication or abnormalities were noted while the horses were under general anesthesia. No intravascular injections were noted in the study, but some contrast was found within the vessel wall (3.6% of the injections) on CT examination only. This is likely to be due to some diffusion of the contrast material (liquid phase of the injectate) because the bulk of latex was entirely extravascular. For the horse where latex and contrast material were not retrieved on 2 injections, a complete dissection of the cervical vertebral vessels and brain was conducted to investigate if a vascular embolism could have occurred. No latex was noted, ruling out the presence of latex embolism in the vertebral vessels and cerebral tissue. Although this complication would be possible in a standing sedated horse and may lead to serious consequences including death, we believe our US-guided technique where a combination of 2D and color Doppler ultrasound is used has the potential to significantly lower this risk compared to other techniques.

Blood was retrieved on two occasions when the stylet was removed before repositioning of the needle. No injectate was found in the vessels and it was assumed that the collateral vessels were punctured and not the main vertebral vessels. The use of ultrasound and color Doppler allowed the operator to identify the relevant vascular structures and to minimize the risk of inadvertent intravascular injection. This study is the first one to be performed on live horses, allowing more accurate evaluation of this possible complication. Although rare, injectate was noted in the vertebral canal in 1.7% of injections on CT and 3.6% of injections on dissection using the technique described herein. Even in those cases, the bulk of latex was always noted outside of the vertebral canal, indicating that the tip of the needle had remained outside of the vertebral canal but some injectate migrated inside the canal during/after injection. As latex was noted in the nerve sheath in 11% of the injections, we suspect that a possible route for the injectate to reach the vertebral canal is by migration from the nerve sheath to the epidural space, as both structures are contiguous. Direct transforaminal diffusion into the epidural space is also a possibility. CT examination allowed us to evaluate the precise localization of the liquid phase of the injectate (contrast material) while dissection allowed us to localize the solid phase of the injectate (latex). Transforaminal diffusion could explain why the presence of injectate into the vertebral canal was slightly more frequent on CT than on dissection. The migration or diffusion of injectate into the vertebral canal should be considered as a therapeutic risk more than a benefit. As shown by Tourzot-Jourde et al., injection more proximally on the nerve and possibly with higher volume are likely to lead to more risk of reaching the epidural space (14). This is a potentially serious problem in equine sports medicine because particulate corticosteroids such as methylprednisolone acetate, triamcinolone acetonide and betamethasone acetate, are commonly used to address chronic pain/inflammation as they last longer at the injection site. Serious adverse events when used for axial spine injections in humans have been reported, including blood vessel injury or spasm, or even embolization through vessels causing spinal cord infarction and necrosis (16–18). Light microscopy studies have demonstrated that particles in these steroid preparations are either larger than red blood cells or can form aggregates larger than red blood cells. Methylprednisolone acetate has a significantly higher percentage of large particles and may occlude vessels. Triamcinolone acetonide preparations have an intermediate particle size and betamethasone acetate has the smallest particle size of the particulate steroids (19). Although this was beyond the scope of our study, but considering the potential (low) risk of diffusion/migration toward the vertebral canal identified with the UG injection technique described here as well as the inadvertent risk of intravascular injection with any technique, dexamethasone phosphate, a non-particulate corticosteroid, may be preferred to other particulate steroids for treatment of radiculopathy.

The main limitation of the study was the fact that horses were injected under general anesthesia compared to clinical cases where the procedure will be performed standing with sedation. This allowed easier positioning and eliminated most risks of the horse moving or reacting during the procedure. Now that accuracy and safety have been assessed with satisfactory results, the next step would be to perform this technique on live conscious horses. The population of horses was only composed of older horses with relatively poor muscling of the neck. This could have potentially allowed easier imaging of the IVF and ventral ramus of the cervical nerve root compared to well-muscled athletic horses. Only 5 horses of the same breed and size were used in the study, limiting the effect of anatomical variations that could have happened in a larger and more diverse group of horses. Another limitation is the use of only one type of injectate (contrast-latex mix) with relatively small volume (1.5 ml). As discussed above, the focus of the study was on the accuracy and safety of needle insertion and injection. Using larger volumes with different viscosities could affect the spreading effect of the injectate both positively (higher success rate) and negatively (spread to non-targeted structures e.g., vertebral canal). Finally, different degrees of operator experience or needle diameter were not tested in the present study, which could have provided additional valuable clinical information.

## Conclusion

Injection of the cervical nerve roots of horses can be performed accurately using the technique described in this study, regardless of the location (C3-C8). Direct contact between the nerve root and the injectate can be expected in ~75% of the cases in anesthetized horses. Injection in the close vicinity (< 5 mm) of the nerve root can be expected in ~25% of the cases, likely reaching the nerves later by diffusion. Based on the relatively rare occurrence of intraneural injection (11%), intravascular injection (none performed in the lumen but 3.6% diffusion in the vessel wall) or intravertebral canal injection (3.6%), we can anticipate that injecting a low volume of non-particulate corticosteroids would be relatively safe in standing sedated horses affected by radiculopathy, assuming the procedure is performed by an experienced operator familiar with the IVF ultrasono-anatomy and the technique described. Further studies are warranted to assess the performance and safety of this technique in standing sedated horses, clinically affected by radiculopathy.

## Data availability statement

The raw data supporting the conclusions of this article will be made available by the authors, without undue reservation.

## Ethics statement

The animal study was reviewed and approved by Institutional Animal Care and Use Committee of the Equine Veterinary Medical Center, a member of Qatar Foundation, Doha, Qatar, under the protocol number EVMC- 2020-1131.

## Author contributions

GF: organizing and conducting the experiments, interpreting and analyzing the results, writing, and revising the manuscript. GA, JJ, and EP: organizing and conducting the experiments and revising the manuscript. CH and EA: organizing and conducting the experiments. SP: interpreting and analyzing the results, writing, and revising the manuscript. FD: hypothesis generation and experimental design, organizing and conducting the experiments, interpreting and analyzing the results, writing and revising the manuscript, and founding. All authors contributed to the article and approved the submitted version.

## Funding

The Intramural Grant Program of the Equine Veterinary Medical Center, Member of Qatar Foundation (Grant Number RG18_FD2 to FD) has funded this research project, including the open access publication fees.

## Conflict of interest

Author SP is employed by Puchalski Equine Inc. The remaining authors declare that the research was conducted in the absence of any commercial or financial relationships that could be construed as a potential conflict of interest.

## Publisher's note

All claims expressed in this article are solely those of the authors and do not necessarily represent those of their affiliated organizations, or those of the publisher, the editors and the reviewers. Any product that may be evaluated in this article, or claim that may be made by its manufacturer, is not guaranteed or endorsed by the publisher.
